# Environmental Sustainability Evaluation of Iron Oxide Nanoparticles Synthesized via Green Synthesis and the Coprecipitation Method: A Comparative Life Cycle Assessment Study

**DOI:** 10.1021/acsomega.0c05246

**Published:** 2021-05-03

**Authors:** David Alfonso Patiño-Ruiz, Samir Isaac Meramo-Hurtado, Ángel Dario González-Delgado, Adriana Herrera

**Affiliations:** † Programa de Doctorado en Ingeniería, Grupo de Nanomateriales e Ingeniería de Procesos Asistida por Computador, Universidad de Cartagena, Cartagena 130010, Colombia; ‡ Departamento de Ingeniería Químmica, Grupo de Investigación Tecnológico Ontare, Universidad EAN, Bogotá 111311, Colombia; § Programa de Ingeniería Química, Grupo de Nanomateriales e Ingeniería de Procesos Asistida por Computador, Universidad de Cartagena, Cartagena 130010, Colombia

## Abstract

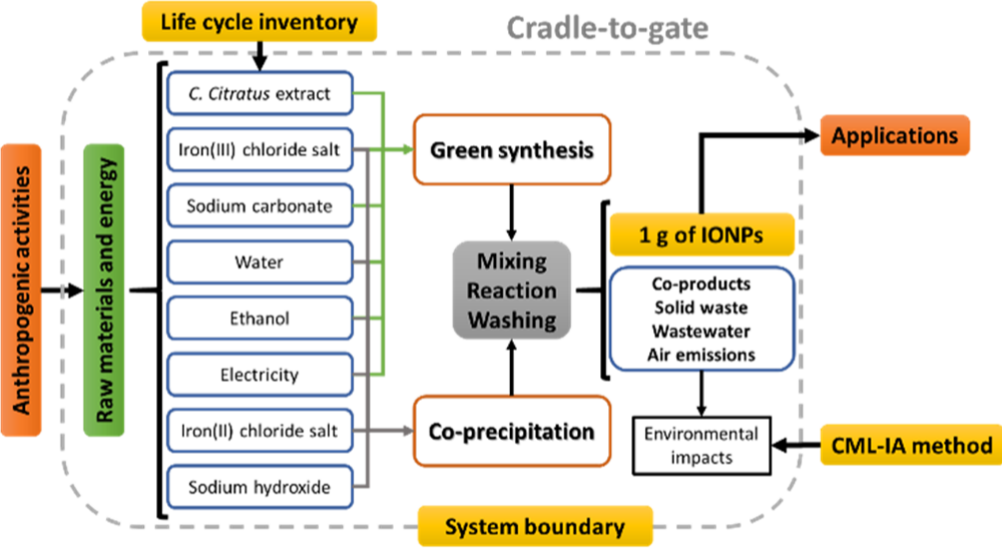

Green synthesis, based on green chemistry, is replacing the traditional methods, aiming to contribute with an enhanced environmental sustainability, which can be achieved using nontoxic compounds from biological resources, such as natural extracts from plants. In this study, the life cycle assessment (LCA) of iron oxide nanoparticles prepared through the green synthesis and the coprecipitation method is reported by following a cradle-to-gate approach. The LCA allowed quantifying and normalized the environmental impacts produced by the green synthesis (1.0 × 10^–9^), which used a *Cymbopogon citratus* (*C. citratus*) extract and sodium carbonate (Na_2_CO_3_). The impacts were also determined for the coprecipitation method (1.4 × 10^–8^) using the iron(II) salt precursor and sodium hydroxide (NaOH). The contribution of *C. citratus* extract and Na_2_CO_3_ as the precursor and pH-stabilizing agents, respectively, was compared regarding the iron(II) and NaOH compounds. Environmental sustainability was evaluated in human toxicity, ecosystem quality, and resource depletion. The major environmental contribution was found in the marine aquatic ecotoxicity (7.6 × 10^–10^ and 1.22 × 10^–8^ for green synthesis and the coprecipitation method) due to the highest values for ethanol (3.5 × 10^–10^) and electricity (1.4 × 10^–8^) usage since fossil fuels and wastewater are involved in their production. The *C. citratus* extract (2.5 × 10^–12^) presented a better environmental performance, whereas Na_2_CO_3_ (4.3 × 10^–11^) showed a slight increase contribution compared to NaOH (4.1 × 10^–11^). This is related to their fabrication, involving toxic compounds, land occupation, and excessive water usage. In general, the total environmental impacts are lower for the green synthesis, suggesting the implementation of environmentally friendlier compounds based on natural sources for the production of nanomaterials.

## 1 Introduction

Nanomaterials have gained significant attention in fields such as medicine^1^ and environmental remediation,^2^ outstanding the application of iron oxide nanoparticles (IONPs). The IONPs present excellent physicochemical properties, including superparamagnetism, biodegradability, biocompatibility,^3^ high surface area, and stability.^4^ Accordingly, increased demand for IONPs suggests changes in the traditional chemical, physical, and biological synthesis,^5^ aiming to improve the cost-effectiveness, environmental sustainability, and manufacturing process.^6^ Alternative methods based on green chemistry arise to overcome these limitations and optimize the production of IONPs with superior environmental performance,^7^ and more efficient processes.^8^ The wide use of nanomaterials for environmental remediation includes the removal, stabilization, and degradation of different organic and inorganic contaminants such as heavy metals and organic matter.^9^ Therefore, the potential environmental impacts of nanomaterials, especially, iron-based nanoparticles, have to be considered and evaluated, which can affect different organisms and microorganisms in a large variety of ecosystems.^10^

Green chemistry, defined by Anastas and Warner in 1998 as the design of environmentally friendly chemical processes and products,^11^ follows 12 principles summarized in the minimization of hazardous compounds and the generation of residues.^12^ Along with sustainable chemistry, green chemistry improves the efficiency related to the use of natural resources, contributing from social, economic, and environmental perspectives.^13^ The implementation of greener technologies aims to reduce the pollution released into the environment, increasing the energy efficiency of the process. This is addressed from an analytical life-cycle approach,^14^ allowing to address strategies, involving environmental management systems, circular economy, and industrial ecology.^15^ Green analytical chemistry is a tool used for the scientific community, involving chemists and engineers, to implement the principles toward the synthesis, processing, analysis, and end-of-life of products.^16^ New techniques and methods, such as green synthesis, have to be alternatives for reducing and eliminating hazardous compounds and wastes, aiming for environmental sustainability at the laboratory and industrial scale.^17^

Accordingly, the green synthesis of nanomaterials avoids the use of organic solvents, surfactants, reducing agents, and stabilizers by replacing them with widely available biological resources.^18^ Among these resources, natural extracts play an important role due to the high phytochemical content, acting as reducing, capping, and stabilizing agents.^19^ These phytochemicals are nontoxic and rich in polyphenols,^20^ promoting the growth of the IONPs,^21^ and affecting the particle size, distribution, and morphology.^19^ Several studies have reported the use of phytochemicals from different biological sources, including *Cucurbita moschata* leaves, *Beta vulgaris* stalks,^21^*Ficus carica* dried fruit,^19^*Lantana camara* flowers,^7^*Moringa oleifera* fruit/leaves,^22^ and *Stachys lavandulifolia* herbal tea.^23^

Although green synthesis is replacing traditional methods, the lack of information about environmental sustainability and human health impacts represents a great challenge.^24^ The implementation of natural extracts reduces the use of chemical compounds, expecting to contribute with less environmental impacts. However, the production of natural extracts at a large scale involves other types of processes, in which toxic chemical compounds are widely used, such as fertilizers and pesticides. These processes usually require large amounts of freshwater, high energy consumption, and fossil fuels, promoting anthropogenic activities for wastewater treatment, energy generation, and oil refining.^24^ Therefore, the use of natural extracts may not mitigate environmental impacts completely. Thus, other factors also need to be addressed, such as energy consumption and resource depletion.^25^ The environmental impacts can be evaluated from a cradle-to-gate approach, using life cycle assessment (LCA) to categorize and quantify impacts.^26^ This approach is associated with the life cycle of the IONPs, including production stages (raw materials, reactions, and purification), as well as the application and final disposition in case of a cradle-to-grave analysis.^27^ The LCA considers early phases, such as the extraction and fabrication of the raw materials, operating conditions during the production process, and the anthropogenic activities for recycling the product.^28^ However, the absence of information and characterization factors is limited and complex when the processes are recent, providing uncertainty and low accuracy in the LCA results since the environmental assessment of nanomaterials production has been scarcely investigated.^5^ Moreover, the LCA does not provide impacts in terms of speed (variables over time).

The LCA is widely applied to new products, technologies, and processes, aiming to determine environmental sustainability^29^ and establish environmental policy regulations through decision-making tools.^27^ As a robust tool, the LCA aims to reduce the uncertainty in environmental sustainability and achieve the quantification of the impacts in input and output streams within the production process of nanomaterials.^30^ The lack of information related to the compounds used as raw materials reduces the accuracy and availability of the life cycle inventory (LCI) to determine the potential environmental risks.^31^ Additionally, the LCA considers the environmental impacts produced during the application, which is currently a requirement for the large-scale production of nanomaterials with enhanced performance.^32^ According to the authors’ best knowledge, the LCA has been scarcely applied to iron-based nanomaterials. Some studies have reported on the green synthesis of zero-valent iron nanoparticles using *Parthenocissus tricuspidata*,^33^ and their utilization for *in situ* environmental remediation.^34^ Additionally, the comparison of traditional synthesis routes to obtain magnetic nanocomposites,^5^ and the acquisition of raw materials for the production of functional magnetite nanoparticles is reported.^35^ The literature also reports the application of LCA to evaluate the production of other types of nanomaterials, such as silver/graphene oxide,^26^ zinc oxide,^30^ titanium dioxide, zirconium dioxide, and lithium/iron/phosphate nanoparticles.^31^ The environmental impacts are commonly associated with the use of metal/metal oxide precursors as the raw materials, representing the major contribution attributed to the energy-intensive processes, such as mining and refining activities.^36^

This study reports the LCA of the green synthesis of functional IONPs, aiming to quantify the environmental impacts using more environmentally friendly compounds. The LCA has been scarcely applied to the green synthesis, outstanding those implemented to produce functional IONPs using natural extracts. Table 1 shows the previous LCA studies performed for the green production of nanomaterials, including the traditional methods. Here, the *Cymbopogon citratus* (*C. citratus*) extract and sodium carbonate (Na_2_CO_3_) were used to replace the iron(II) salt precursor and the hydroxide compounds, of which the latter two are considered as less environmentally friendly. *C. citratus* was used to reduce the iron(III) to iron(II), whereas Na_2_CO_3_ was a pH-stabilizing agent. Na_2_CO_3_ is well known as a simple salt compound with a small carbon footprint and presents good engineering performance in terms of cost effectiveness^37^ and health risks.^38^ Additionally, the IONPs were synthesized *via* the coprecipitation method to compare the environmental impacts with those produced from the green synthesis. The coprecipitation method consisted of the reaction between iron(III) and iron(II) salt precursors, along with sodium hydroxide (NaOH) as the pH stabilizer agent. For the first time, an LCA study was conducted to evaluate the environmental sustainability of the process when a natural extract and simple salt compound are used as the raw materials.

**Table 1:** LCA Studies Related to the Production of Nanomaterials *via* Green Synthesis and Other Traditional Methods

nanomaterial	technique	raw material
**functional iron oxide (this study)**	green synthesis	iron(III) chloride hydrated, *C. citratus* extract, sodium carbonate
	coprecipitation	iron(III) chloride hydrate, iron(II) chloride hydrated, and sodium hydroxide
**iron oxide**(39)	coprecipitation and green synthesis	iron(III) chloride and l-gluthaione
sterically stabilized**, PEI, oleic acid, andsilica dioxide-coatediron oxide**^5^	coprecipitation and hydrolysis/condensation	iron(III) chloride hydrate, iron(II) sulfate hydrate, hydrochloric acid, tetramethylammonium hydroxide, polyethylenimine, oleic acid, Igepal CO-520, cyclohexane, ammonium hydroxide, and tetraethyl orthosilicate
**carbon dots**(40)	hydrothermal and microwave-assisted	citric acid and urea
**silver oxide**(41)	green synthesis	silver nitrate, glucose, and food-grade corn starch
**silver oxide**(42)	chemical reduction and green synthesis	silver nitrate, trisodium citrate, sodium borohydride, ethylene glycol, and soluble starch
	flame spray pyrolysis	silver octadecanoate
**silica dioxide**(43)	wet synthesis	styrene, poly(vinylpyrrolidone), potassium persulfate, tetraethyl orthosilicate, and ammonium hydroxide
**titanium dioxide**(44)	chemical, physical, and biological routes	titanium oxysulfate, and titanium tetrachloride, titanium isopropoxide, titanium tetrabutoxide
**zinc oxide**(30)	microwave	zinc nitrate hexahydrate and hexamethylenetetramine

## 2 Results and Discussion

**Figure 1:**
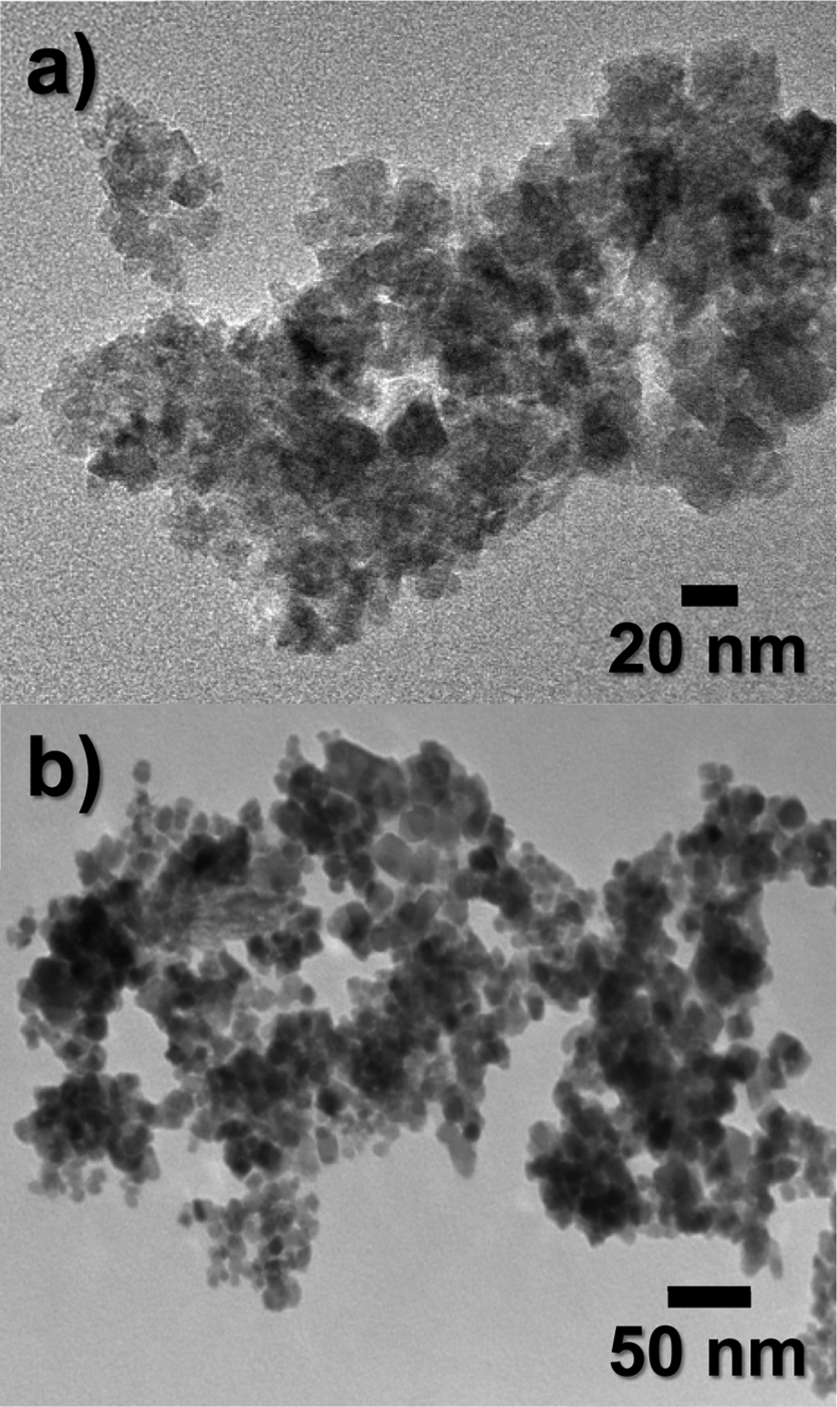
TEM images of the IONPs synthesized *via* (a) green chemistry and (b) the coprecipitation method.

Figure 1a,b shows the transmission electron microscopy (TEM) images of the IONPs synthesized *via* the green chemistry and the coprecipitation method, respectively, displaying regular crystalline structures. Patiño-Ruiz reported a complete physicochemical characterization of the as-produced IONPs *via* the coprecipitation method using iron(III) and iron(II) salt solutions,^45^ and *C. citratus* extract for the green synthesis.^46^ From Figure 1a, an average diameter size of around 9 ± 4 nm was determined,^46^ whereas as shown in Figure 1b, the IONPs presented a similar average of 10 ± 4 nm.^47^ The crystal structure was corroborated corresponding to the planes of superparamagnetic iron metal oxides (Fe_3_O_4_ and γ-Fe_2_O_3_ phases) obtained from both methods. Moreover, a dense agglomeration was observed due to the strong dipole–dipole interaction between nanoparticles, which is typical for IONPs with a high surface reactivity.^46,47^

The total environmental impacts of each method performed at the laboratory scale are shown in Table 2. The contribution of the green synthesis in each category was significantly lower compared to that of the coprecipitation method. Although the procedure and the production yield at the laboratory scale are similar, the coprecipitation method showed major environmental disadvantages. Among the impact categories, the marine aquatic ecotoxicity (MAE) exhibited the highest contribution with values up to 8.9 × 10^4^ and 1.4 × 10^6^ kg 1,4-DB equiv for the green synthesis and the coprecipitation method, respectively. Meanwhile, the lowest contribution in each method was observed to be 5.9 × 10^–6^ and 3.7 × 10^–4^ kg CFC-11 equiv, respectively.

**Table 2:** Total Environmental Impacts of the Methods Performed at a Laboratory Scale, Considering an IONP Production Yield of 4.2 g

impact category	coprecipitation	green synthesis
AD	1.5 × 10^–3^	4.9 × 10^–4^
AD-ff	9.6 × 10^3^	2.4 × 10^3^
GWP	8.2 × 10^2^	1.3 × 10^2^
ODP	3.7 × 10^–4^	5.9 × 10^–6^
HT	5.5 × 10^2^	1.2 × 10^2^
FAE	5.7 × 10^2^	4.4 × 10^1^
MAE	1.4 × 10^6^	8.9 × 10^4^
TE	1.8 × 10^0^	3.3 × 10^–1^
PO	1.6 × 10^–1^	7.9 × 10^–2^
TA	3.3 × 10^0^	4.4 × 10^–1^
EP	3.7 × 10^0^	1.9 × 10^–1^

In the case of the normalized environmental impacts summarized in Table 3, the contribution with the highest impacts was also for the MAE category in both methods. These results suggest a better environmental performance for the green synthesis, mainly attributed to the use of the *C. citratus* extract and Na_2_CO_3_, instead of iron(II) precursor salt and NaOH. Although few studies have reported the LCA of synthesis routes for nanomaterial production, especially for IONPs, the environmental impacts are considerably lower compared to analogue synthesis routes (see Table 4).

**Table 3:** Normalized Environmental Impacts (Normalization Using CML-IA) for the Production of 1 g of IONPs

impact category	coprecipitation	green synthesis
AD	1.8 × 10^–11^	5.8 × 10^–12^
AD-ff	3.1 × 10^–10^	7.5 × 10^–11^
GWP	1.6 × 10^–10^	2.5 × 10^–11^
ODP	4.1 × 10^–12^	6.6 × 10^–14^
HT	7.1 × 10^–11^	1.5 × 10^–11^
FAE	1.1 × 10^–9^	8.5 × 10^–11^
MAE	1.2 × 10^–8^	7.6 × 10^–10^
TE	3.6 × 10^–11^	6.7 × 10^–12^
PO	1.9 × 10^–11^	9.3 × 10^–12^
TA	1.2 × 10^–10^	1.6 × 10^–11^
EP	2.8 × 10^–10^	1.4 × 10^–11^

**Table 4:** Comparison of the Normalized Environmental Impacts between Analogue Synthesis Routes Reported in the Literature and Those Developed in This Study^a^

	literature^39^		this study	
impact category	CS	GS	CP	GS
AD			1.8 × 10^–11^	5.8 × 10^–12^
AD-ff	5.0 × 10^–3^	8.0 × 10^–2^	3.1 × 10^–10^	7.5 × 10^–11^
GWP	3.0 × 10^–3^	4.0 × 10^–2^	1.6 × 10^–1^0	2.5 × 10^–11^
ODP			4.1 × 10^–12^	6.6 × 10^–14^
HT	7.0 × 10^–4^	6.0 × 10^–4^	7.1 × 10^–11^	1.5 × 10^–11^
FAE	1.0 × 10^–2^	9.0 × 10^–2^	1.1 × 10^–9^	8.5 × 10^–11^
MAE	9.0 × 10^–5^	6.0 × 10^–4^	1.2 × 10^–8^	7.6 × 10^–10^
TE	1.0 × 10^–4^	4.0 × 10^–2^	3.6 × 10^–11^	6.7 × 10^–12^
PO	8.0 × 10^–5^	6.0 × 10^–5^	1.9 × 10^–11^	9.3 × 10^–12^
TA	7.0 × 10^–5^	5.0 × 10^–5^	1.2 × 10^–10^	1.6 × 10^–11^
EP			2.8 × 10^–10^	1.4 × 10^–11^

a (CS = conventional synthesis, GS = green synthesis, and CP = coprecipitation)

**Figure 2:**
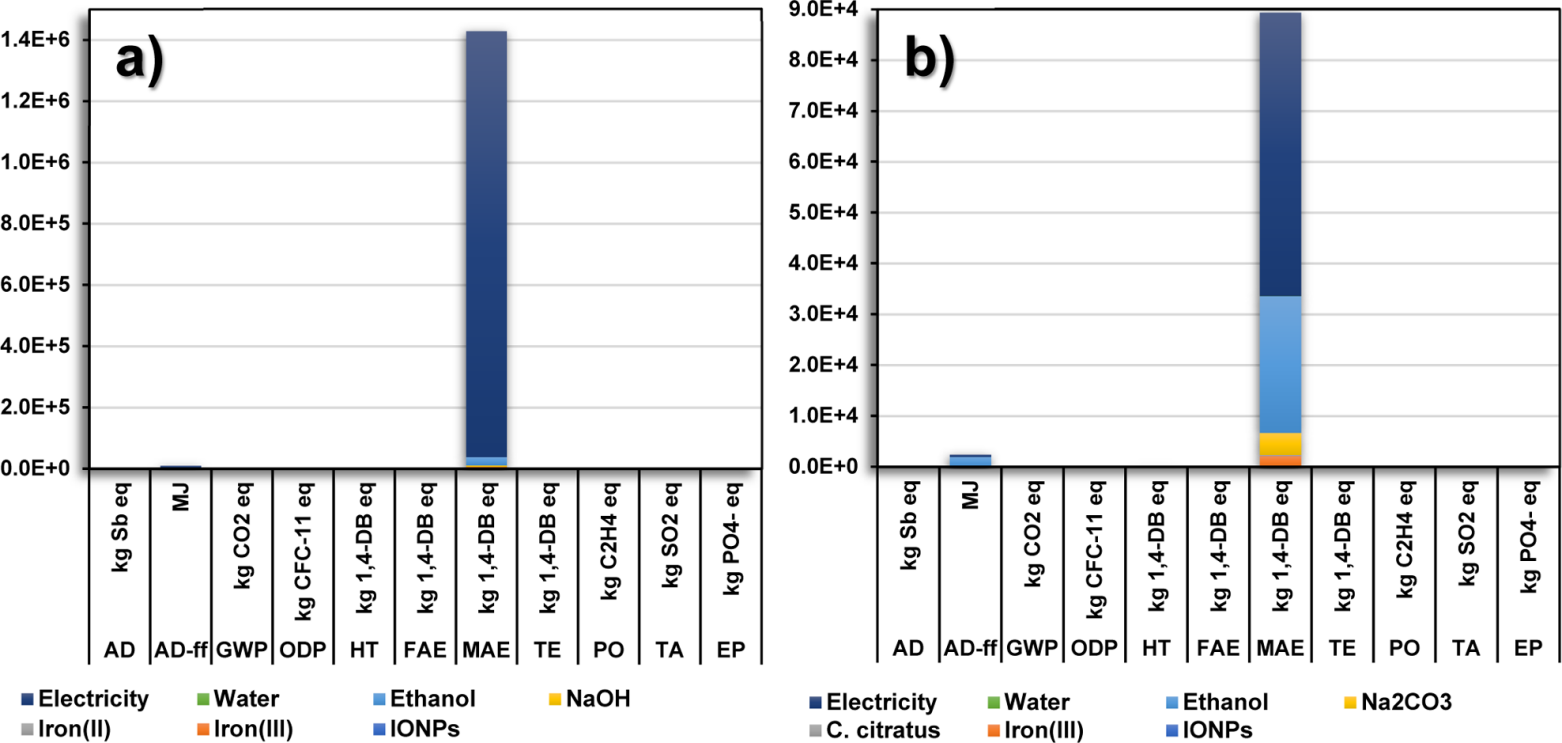
Distribution of the environmental impacts for each inventory item required for the production at a laboratory scale *via* (a) the coprecipitation method and (b) green synthesis.

The environmental impact distributions for the green synthesis and coprecipitation method are displayed in Figure 2a,b, respectively. The contribution was mainly attributed to the use of ethanol and electricity during IONP preparation. In the case of the environmental impact distribution for the coprecipitation method in Figure 2a, electricity generation showed the highest contribution for the MAE. This result suggests that at a laboratory scale, the coprecipitation method requires more massive amounts of energy compared to the green synthesis, which corroborates the total environmental impacts for MAE shown in Table 2. Figure 2b shows a slight contribution in the AD-ff, considering that the fabrication of ethanol and electricity generation requires fossil fuel resources.^30^ Moreover, impacts in the MAE category are attributed to the extensive use and disposal of ethanol for the purification of the IONPs, along with the effluents containing toxic compounds from the electricity generation that is released into the water resources. Among these toxic compounds are commonly found heavy metals, sulfuric compounds, and polycyclic aromatic hydrocarbons. Additionally, iron(III) and Na_2_CO_3_ contributed to the MAE by disposing of a vast volume of wastewater from the mining activities since these compounds require minerals for their fabrication.^26^

**Figure 3:**
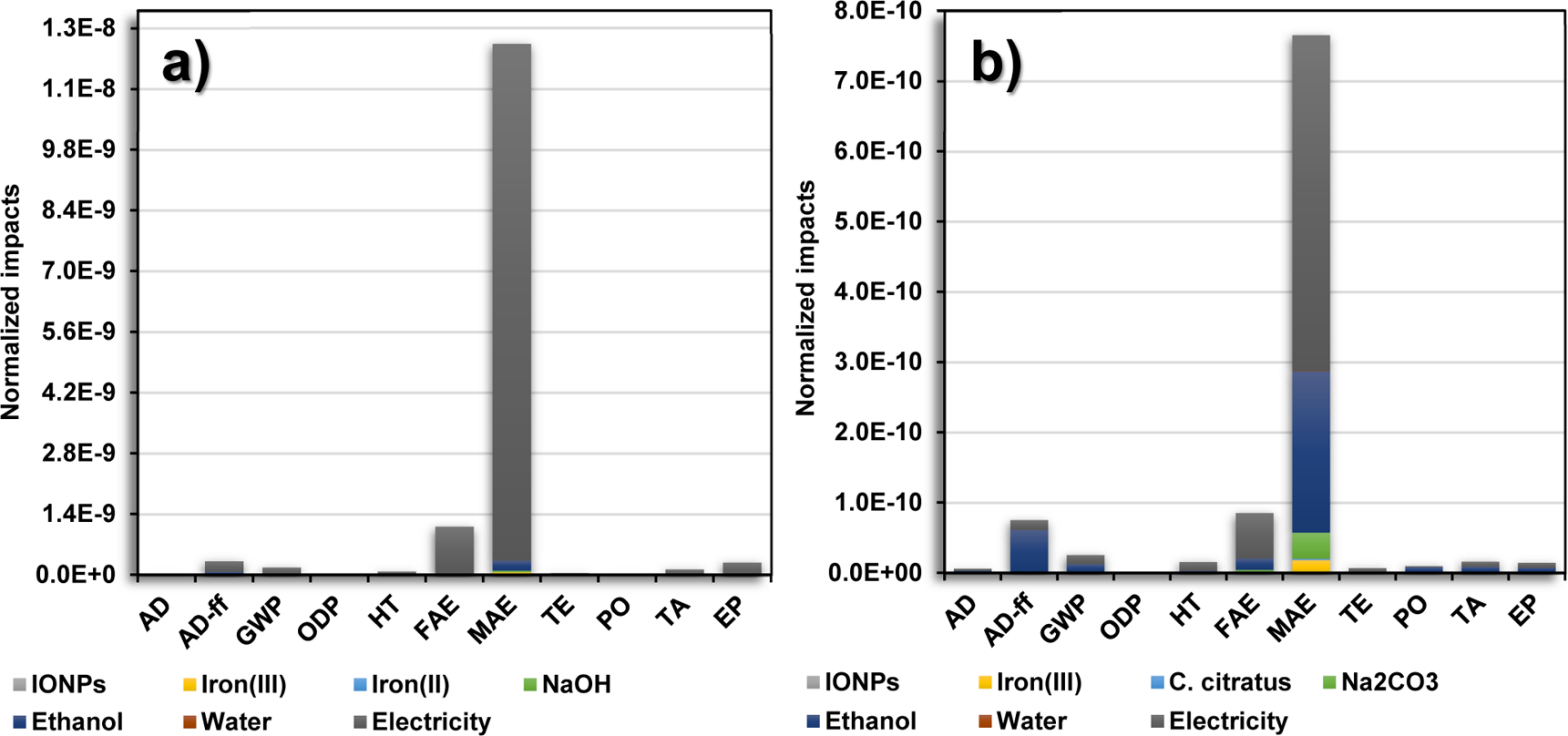
Distribution of the normalized environmental impacts (normalization using CML-IA) for each inventory item required for the production of 1 g of IONPs *via* (a) the coprecipitation method and (b) green synthesis.

The normalized environmental impacts for the production of 1 g of IONPs are displayed in Figure 3a,b. In both methods, the contribution to the impact categories was extended, allowing the identification of the effects after scaling up the production of IONPs. The most affected category is still the MAE, followed by the AD-ff, GWP, and FAE. The coprecipitation method distribution is illustrated in Figure 3a, observing the contribution to several impact categories, highlighting the electricity generation. The normalized results showed a reduction in the environmental impact gap between green synthesis and the coprecipitation method. However, the latter method presented a significantly lower environmental performance with higher impacts in all the categories, in which the lowest and highest contributions were 4.1 × 10^–12^ kg CFC-11 equiv and 1.2 × 10^–8^ kg 1,4-DB equiv in the ODP and MAE categories (see Table 3), respectively. The incorporation of *C. citratus* extract and Na_2_CO_3_ promotes environmental improvement, showing a better performance interestingly from an energetic point of view. The environmentally friendlier compounds used in the green synthesis showed promising results compared to other green methods reported in the literature.^5,48^

Similar results were observed for the green synthesis, as shown in Figure 3b, indicating a slight contribution in the AD, HT, TE, PO, TA, and EP categories. As was already mentioned, the contribution in all categories was attributed mainly to the use of ethanol and electricity. The production of these compounds involves fossil fuel extraction, refining, and combustion, promoting the release and emission of solid, liquid, and gaseous wastes into the environment.^48^ The contribution of iron(III) was related to a possible release of iron ions,^49^ and the large amount used for the reduction to iron(II), considering that the latter compound was replaced by the *C. citratus* extract in the green synthesis. However, the low value was related to the iron(III) salt precursor composition since it was composed of chlorides instead of more toxic compounds such as sulfates.^35^ In the case of the Na_2_CO_3_, the contribution is specifically related to the mining process for its fabrication. Na_2_CO_3_ is considered green due to its lesser corrosive and toxic effects in humans and ecosystems,^38^ compared to other pH-stabilizing agents such as hydroxides.

**Figure 4:**
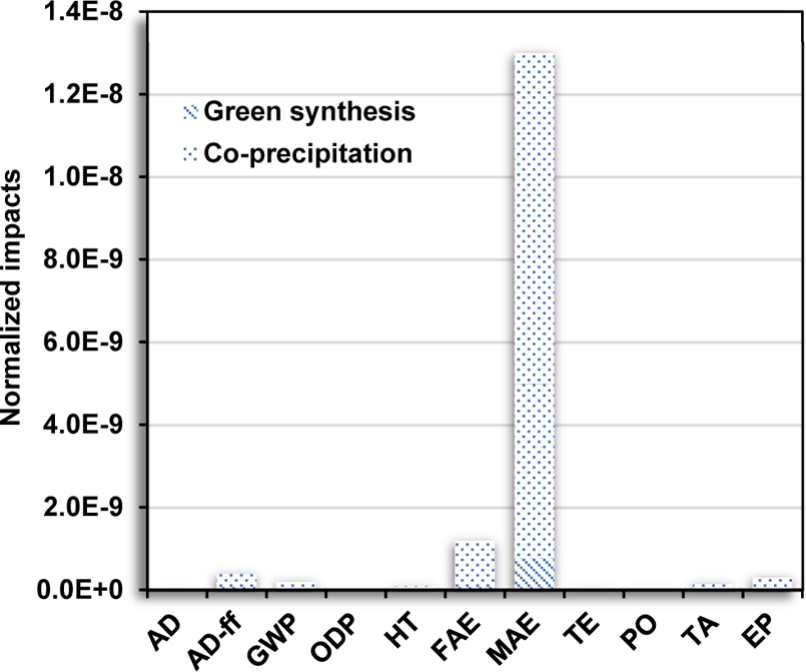
Comparative analysis of the normalized environmental impacts (normalization using CML-IA) in both methods, considering the production of 1 g of IONPs.

A comparative analysis between the green synthesis and the coprecipitation method is described in Figure 4. In general, the normalized environmental impacts for the coprecipitation method were considerably higher than the green synthesis in all the categories. Here, the impacts of green synthesis were insignificant. Considering that the process was similar for both methods, the coprecipitation method contributed to a greater magnitude in the impact categories related to the AD-ff, GWP, FAE, MAE, TA, and EP.

As previously discussed, the MAE category was predominant, which can be related not only due to the high consumption of ethanol and energy but also due to the use of the iron(II) salt precursor and the NaOH stabilizer agent. The ethanol, iron(II), and NaOH compounds involve different anthropogenic activities for their production. Therefore, it implies higher impacts attributed to energy usage and disposal of industrial wastes, typically released into soils and water sources. The production of NaOH represents a potential effect on the AD-ff and TA since the process involves the combination of pure sodium metal with large amounts of water.^35^ Additionally, many of these wastes include organic compounds and heavy metals that can easily migrate from soil to water sources, including groundwater. This explains the contribution in the FAE category, which was the second with the highest impacts. However, the use of greener compounds has to be considered since the environmental impacts tend to decrease significantly, which agrees with the concept related to environmental sustainability in industrial processes.

**Figure 5:**
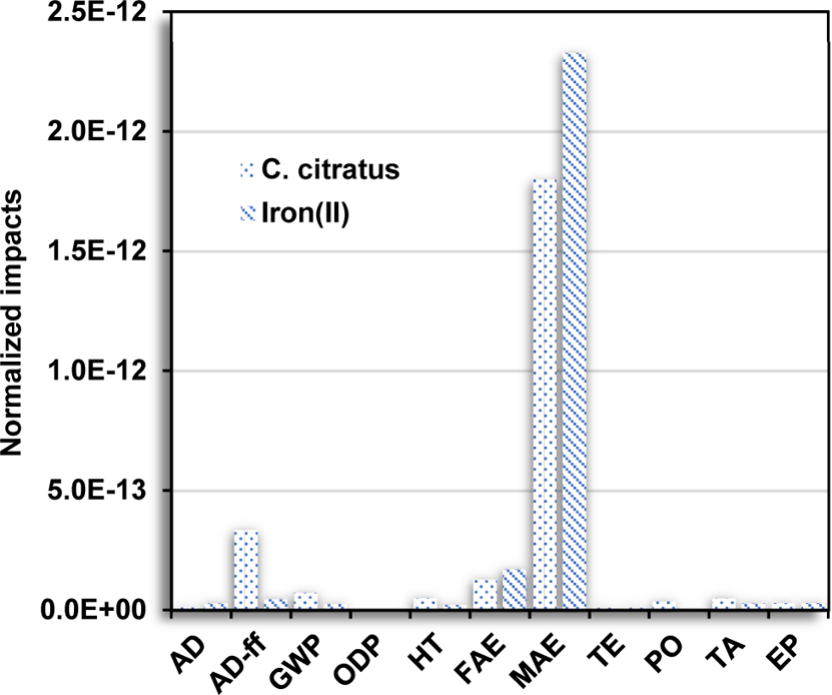
Comparative analysis of the normalized environmental impacts (normalization using CML-IA for the production of 1 g of IONPs) for the use of *C. citratus* and iron(II) in the green synthesis and coprecipitation method, respectively.

The anthropogenic activities require a high demand for fossil fuels, promoting an accelerated depletion and contributing to other categories. Figure 5 shows a comparison of the normalized environmental impacts between the use of *C. citratus* extract and iron(II) salt precursor. According to the CML-IA method for the production of 1 g of IONPs, the use of *C. citratus* extract promotes a reduction in the FAE and MAE categories compared to iron(II). However, a slight significant contribution was observed for AD-ff, GWP, HT, PO, TA, and EP, indicating possible adverse effects attributed to the intensive processes for its production.

The *C. citratus* extract was assumed to be prepared as a lemongrass oil mainly composed of citral and water. However, large-scale production of this extract can involve intensive anthropogenic processes, along with land occupation and transformation. Among the intensive processes, depletion of water and other resources is required, including the lemongrass leaves to extract the oil. The agricultural land occupation and transformation include the use of toxic and corrosive chemicals as pest control methods, which can also contribute to human health deterioration. In general, the environmental impacts are considerably low compared to other green methods, as reported by Marimón-Bolívar and González (2020). Here, glutathione was used as the green compound and reported ecological effects majorly in human toxicity, photochemical oxidation, and fossil depletion with values above 2.1 × 10^–12^, 1.4 × 10^–5^, and 4.2 × 10^–9^, respectively.^39^ Although some impact categories showed a slight major contribution from the *C. citratus* extract, improved environmental performance can be achieved with process intensification and alternative technologies for its production. Meanwhile, the iron(II) fabrication involves anthropogenic activities with considerably higher impacts, such as mining and refining processes for metal extraction. In this study, the iron(II) chloride salt was assumed to react completely, converting into the IONPs and producing NaCl as a valuable coproduct. These assumptions allowed to reduce the impact categories, assuming that no iron or chloride ions were released into the environment.

**Figure 6:**
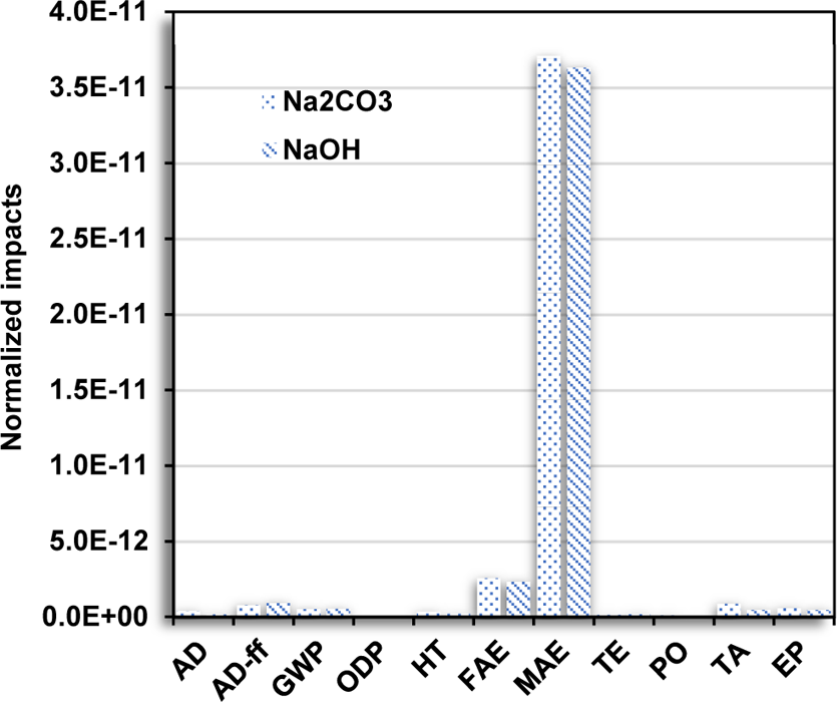
Comparative analysis of the normalized environmental impacts (normalization using CML-IA for the production of 1 g of IONPs) for the use of Na_2_CO_3_ and NaOH in the green synthesis and coprecipitation method, respectively.

The distribution in each impact category considering the environmental contribution due to the use of Na_2_CO_3_ and NaOH is shown in Figure 6. Na_2_CO_3_ can be easily found in the environment from plants in soils and water sources. However, its extraction includes anthropogenic activities such as mining and transportation, promoting an increase in the environmental impacts with similar values compared to those for the use of NaOH. For Na_2_CO_3_ extraction, the burning of plants allows it to be extracted from the ashes, contributing to the AD-ff and GWP categories due to the high consumption of energy. Additionally, Na_2_CO_3_ is also fabricated from a reaction using salt and sulfuric acid, leading to the production of sodium sulfate and hydrochloric acid. Sodium sulfate is then heated in the presence of limestone and coal to produce Na_2_CO_3_ and calcium sulfate as a coproduct. In this latter process, many pollutants are produced and released into the environment, including acids, organic solvents, and carbon dioxide, which contribute to the environmental impacts in GWP, TA, and EP categories.

Although Na_2_CO_3_ is considered to be environmentally friendlier than NaOH and can be found naturally in the environment, the extraction process substantially decreases the environmental performance. Additionally, carbon dioxide is produced during the green synthesis reaction, which was also assumed for the calculation of these results, allowing determination of similar environmental performance between both pH stabilizer agents. A reduction of the environmental impacts can be achieved by implementing technologies for carbon dioxide permeation and storage, as well as the recovery of the NaCl formed with the reaction between Na_2_CO_3_ and iron(II) salt precursor. Although green compounds are considered essential for the environmental sustainability of industrial processes, performing a life-cycle analysis allows establishing optimization procedures to decrease the environmental impacts even more.

**Figure 7:**
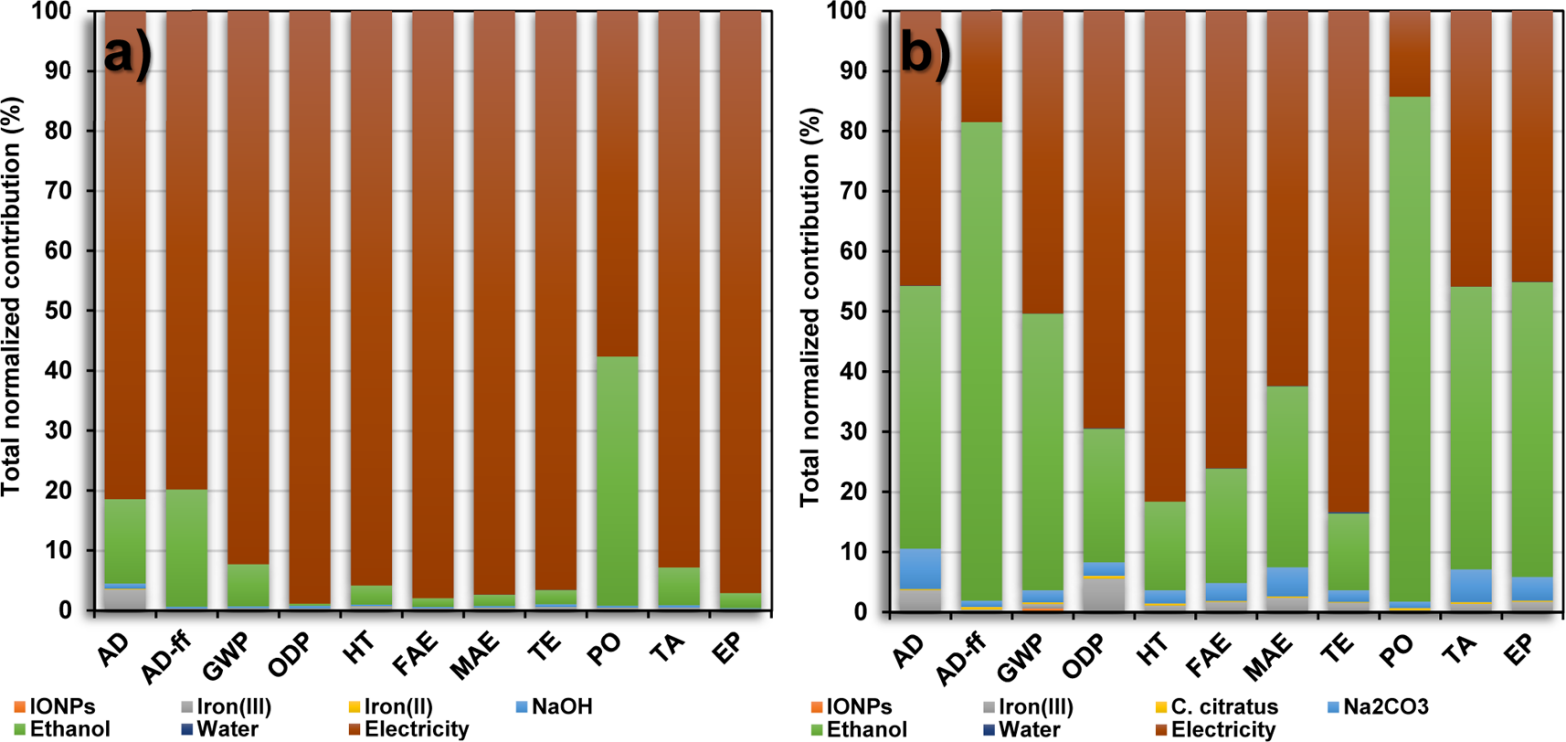
Normalized relative contribution (normalization using CML-IA) of each inventory item during the preparation of 1 g of IONPs *via* (a) the coprecipitation method and (b) green synthesis.

The normalized relative contributions of the green synthesis and the coprecipitation method are shown in Figure 7a,b, respectively. The contribution was determined for each impact category using the CML-IA method to produce 1 g of IONPs. The main environmental weaknesses in both production processes are the use of ethanol and electricity, which occurred in all the impact categories. The highest contributions of ethanol usage were 84 and 42% for the PO category, whereas the electricity usage was 83 and 99% in the TE and ODP categories for the green synthesis and coprecipitation method, respectively. Optimization of both processes needs to be considered, aiming to reduce the environmental impacts in terms of energy and ethanol usage in the separation and purification stages. The reactions were carried out at 85 °C for 1 h in both methods, requiring high energy consumption that cannot be changed, since this is important to promote the growth of the IONPs.

On the other hand, the iron(III) environmental impacts were 6% for the green synthesis, double compared to the 3% of the coprecipitation method. This increase was attributed to the higher amount of the iron(III) salt precursor in the green synthesis, which is required for its reduction to iron(II), and then, the growth of the IONPs. The addition of Na_2_CO_3_, as a pH stabilizer agent, showed a relative contribution of 7%, whereas the use of NaOH produces impacts up to 1%. As discussed in Figure 6, the fabrication of Na_2_CO_3_ demands intensive processes, including carbon dioxide emissions during the main reaction and the waste of coproducts such as NaCl in the wastewater. Moreover, the contribution of the *C. citratus* extract was low to negligible, with less than 1%, allowing determination of its viability for the reduction of iron(III) instead of using the iron(II) salt precursor. According to the CML-IA method, the use of the *C. citratus* extract is considered environmentally friendly and well known as a natural compound.

**Figure 8:**
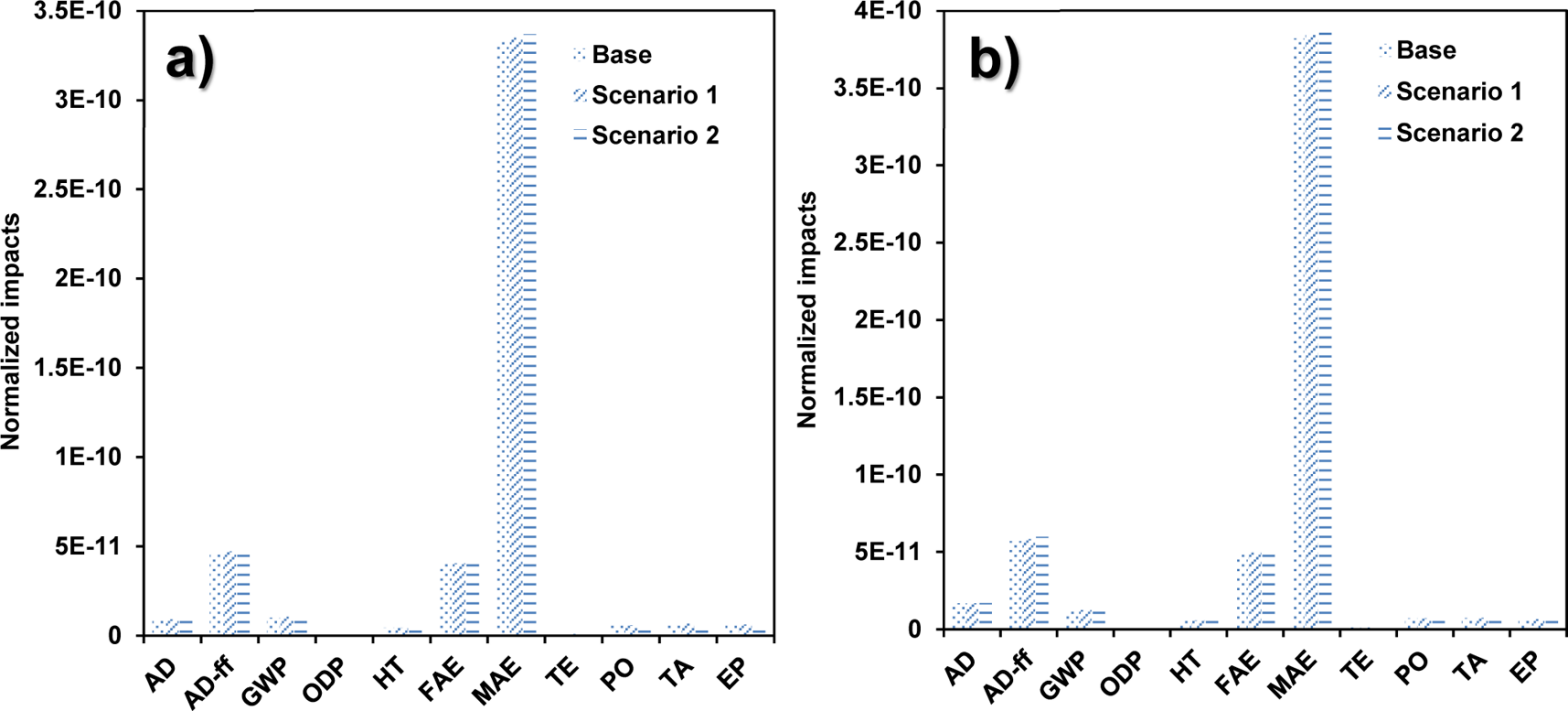
Sensitivity analysis for (a) the coprecipitation method and (b) green synthesis considering electricity in three scenarios.

The sensitivity analysis for the coprecipitation method and green synthesis is displayed in Figure 8a,b. These results allowed analyzing the variation in the environmental impacts for each category when the electricity (energy consumption) is increased by 50 and 100% (scenarios 2 and 3, respectively). Electricity was chosen over the other items in the LCI since those are simpler to control according to the protocol used in this LCA study. The environmental impacts presented a slight increase with the increase in the electricity item from the LCI, which means that there is not a significant contribution regarding the base scenario (actual electricity consumption in this LCA study). The tendency is evident for each impact category, outstanding the contribution for MAE which was previously discussed.

**Figure 9:**
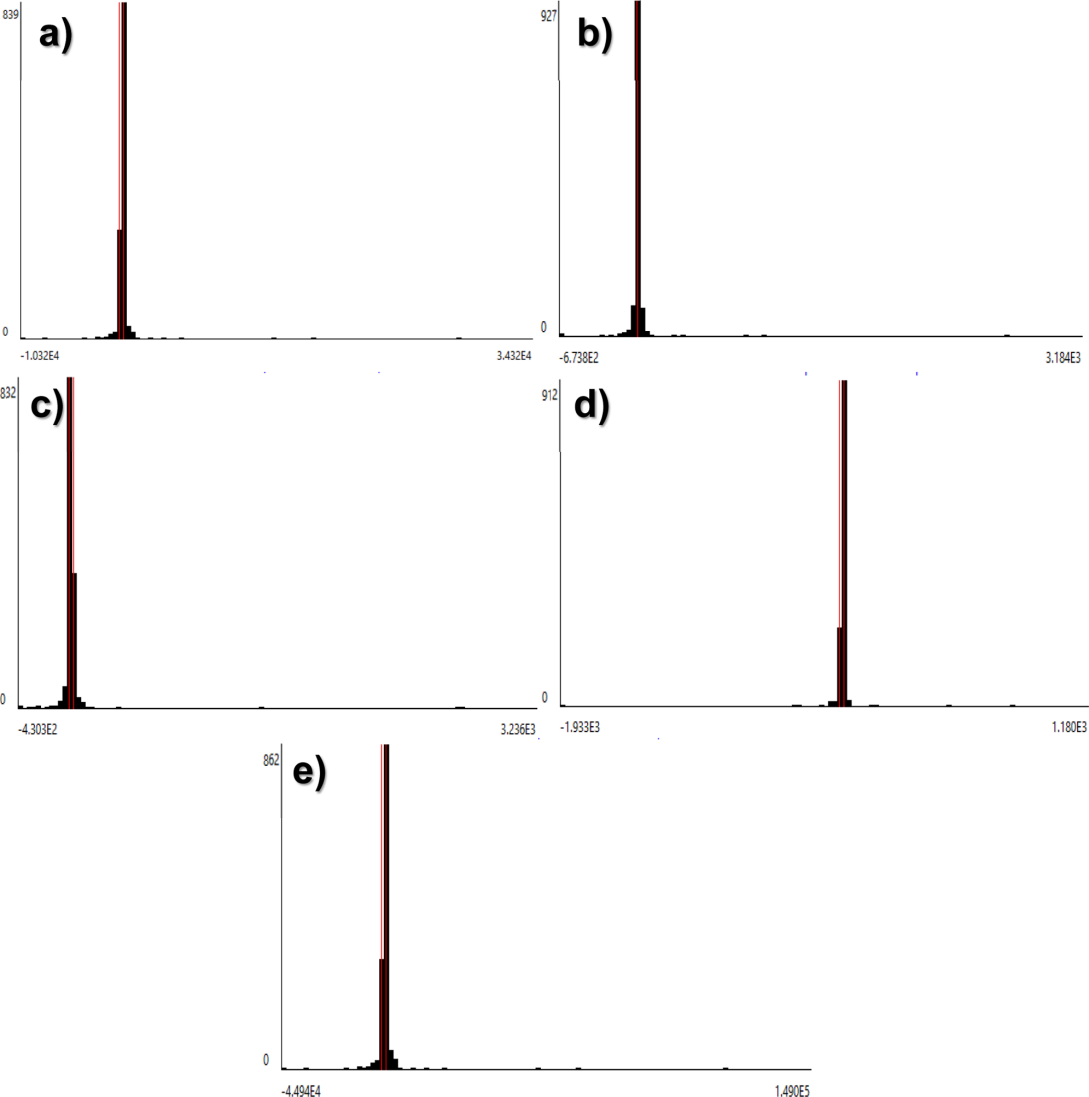
Monte Carlo simulations for green synthesis inventory including (a) ethanol, (b) Na_2_CO_3_, (c) *C. citratus*, (d) iron(III), and (e) water.

**Figure 10:**
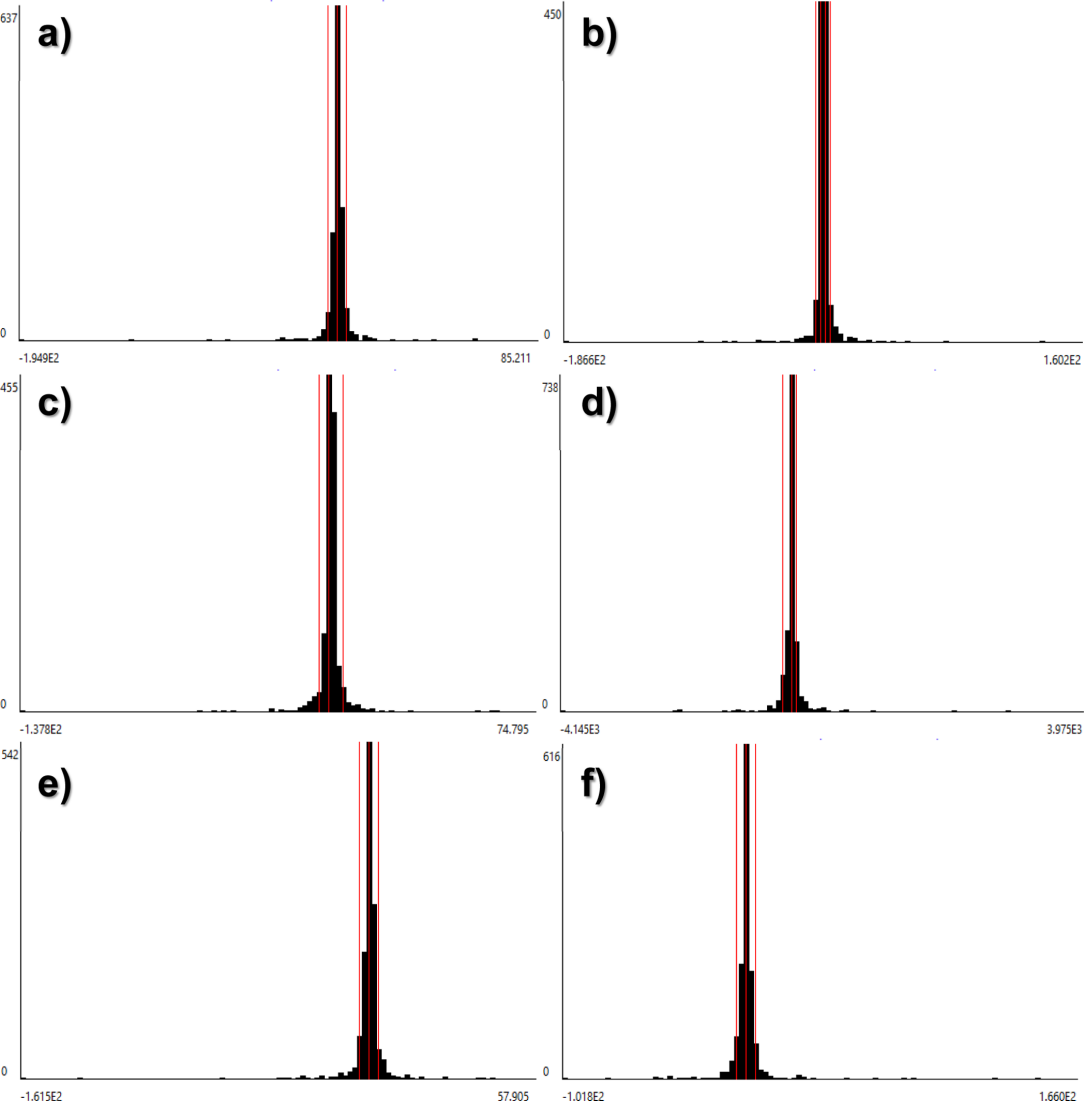
Monte Carlo simulations for the coprecipitation inventory including (a) ethanol, (b) Iron(III), (c) NaOH, (d) electricity, (d) water, and (f) iron(II).

An uncertainty analysis was performed, as shown in Figure 9a–e for the green synthesis, allowing comparison of different values in a probabilistic distribution within a specific uncertainty range. Therefore, the quantitative data meet a certain distribution which confirms data confidence. In this study, 1000 iterations were used and Monte Carlo simulations were made to count the uncertainty influence of the process inventory. It is worth mentioning that normal distribution was used for the input parameters. All the functional units vary around the mean values, but these variations are not very pronounced, indicating a low uncertainty level in process inventory data. In the case of the coprecipitation method, Figure 10a–d shows the uncertainty analysis that was also implemented for verifying the probabilistic behavior of process inventory data. As developed by the green synthesis, a similar approach was implemented in this topology. Normal distribution was assumed for the input parameters in this case study. Functional units vary around the mean values but with higher deviation concerning the mean. In this sense, variation was more evident for iron(III), NaOH, and water. It is also palpable that higher levels of uncertainty were found for green synthesis topology than those reported by the coprecipitation method.

## 3 Conclusions

This study reports the LCA of the green synthesis of IONPs using *C. citratus* extract and the Na_2_CO_3_ stabilizing agent. Moreover, the traditional coprecipitation method was performed using iron(II) and NaOH instead of the *C. citratus* extract and Na_2_CO_3_, respectively, which an LCA allowed to compare the environmental impacts regardless of the green synthesis. In general, the use of ethanol and electricity were the items from the inventory with the highest relative contributions in both methods for a functional unit of 1 g. Here, ethanol had a total relative contribution of 42 and 84% for the coprecipitation method and green synthesis, and the electricity was around 99 and 83%, respectively. These relative contributions were mainly attributed to the implementation of intensive processes required for their fabrication, including the use of fossil fuels and the further disposal of wastewater. In the case of the ethanol, it was extensively used only for the purification of the IONPs and then discarded without being recovered, considering its high value. Hence, an optimization or process intensification can be addressed to recover ethanol and to improve the environmental performance of the processes.

Accordingly, the MAE was the impact category with the major normalized environmental weakness of 1.2 × 10^–8^ and 7.6 × 10^–10^, whereas FAE had the second position with values of 1.1 × 10^–9^ and 8.5 × 10^–11^ for the coprecipitation method and green synthesis, respectively. Moreover, impacts on the AD-ff were also observed in the case of the production at the laboratory scale. These results were even with the use of greener compounds such as *C. citratus* extract and Na_2_CO_3_, which are well known as environmentally friendly since they can be easily found in the environment. However, the production process for their fabrication involves anthropogenic activities, such as excessive use of water, disposal of corrosive and toxic compounds, land occupation, and transformation, among others. A decrease in the environmental performance of the green synthesis occurred due to these anthropogenic activities, but it still presented lower impacts in each category compared to the coprecipitation method. According to these results, green synthesis showed a promising alternative for the production of IONPs, in which the replacement of raw materials with other environmentally friendlier compounds contributes to reduction of the environmental impacts. Although the green synthesis using *C. citratus* extract and Na_2_CO_3_ showed better results and higher viability, the scarcely green methods found in the literature are still a challenge aiming at the deeper comparison. On the other hand, the variation in the uncertainty analysis was more evident for iron(III), NaOH, and water in the LCI, in which higher levels of uncertainty were found for green chemistry topology than those reported by the coprecipitation process. Additionally, future works can be addressed related to the large-scale production of IONPs *via* the green synthesis, including feasibility assessment, “circular economy”, exergetic evaluation, and process simulation. A more complete LCA study can be performed by following a cradle-to-grave approach using other sustainability parameters, including scaled-up production, final disposition, and effects during the application of IONPs.

## 4 Experimental Methods

The environmental impacts of the green synthesis of IONPs were determined through LCA. Additionally, the IONPs were synthesized *via* the coprecipitation method, aiming to compare the resulting environmental impacts regarding the green synthesis. The main differences were the use of a *C. citratus* (*C. citratus*) extract and sodium carbonate (Na_2_CO_3_) in the green synthesis, instead of the typical iron(II) chloride salt and sodium hydroxide (NaOH), generally used for the coprecipitation method. The *C. citratus* extract allowed the reduction of the iron(III) to iron(II), and Na_2_CO_3_ played the role of the pH-stabilizing agent. In this study, the goal and scope were defined, as well as the system boundaries, aiming to provide more accurate results and considering the lack of information in the nanotechnology field. The LCI was established to calculate the environmental contribution in each of the impact categories.

### 4.1 Production of IONPs

#### 4.1.1 Green Synthesis

The green synthesis was conceptualized and developed by our research group.^46^ In this method, *C. citratus* extract and Na_2_CO_3_ were implemented as environmentally friendly compounds, while iron(III) chloride salt was used as the primary iron precursor. Initially, *C. citratus* leaves were pretreated, ground, and added in 800 mL of distilled water at 80 °C. The volume solution was reduced to 100 mL, filtered, and cooled at room temperature. Afterward, a 0.26 M iron(III) chloride solution was prepared using 40 mL of the *C. citratus* extract under 120 rpm of mechanical stirring at 60 °C for 1 h. Then, an additional 0.52 M iron(III) chloride solution was added, adjusting the pH between 10 to 12 with 100 mL of a 0.75 M Na_2_CO_3_ solution. The reaction was carried out at 120 rpm, increasing the temperature up to 85 °C for 1 h. After the reaction, the IONPs were washed three times with distilled water (240 mL each) and once with ethanol (240 mL) by using a centrifuge at 20,000 RFC for 20 min at room temperature. Finally, the IONPs were dried in an oven at 70 °C for 24 h.

#### 4.1.2 Coprecipitation Method

The coprecipitation reaction was carried out considering a 2:1 molar ratio of iron(III) and iron(II) chlorides, respectively.^47^ Here, two 50 mL solutions of 0.52 M iron(III) and 0.26 M iron(II) were prepared individually and then mixed under mechanical stirring and heated up to 85 °C for 1 h. The pH was adjusted by adding 100 mL of 1 M NaOH solution. The purification process was the same one employed for the green synthesis, using washes with distilled water and ethanol, and centrifugation. The IONPs were dried in an oven at 70 °C for 24 h.

### 4.2 LCA of the Green Synthesis and the Coprecipitation Method

According to the international standards ISO 14040 and ISO 14044,^50^ the LCA methodology involved the definition of the goal and scope, system boundaries, the LCI, and the environmental impacts. The LCA was performed using the computational tool SimaPro 9.0.0.49 software. The impact categories were normalized and evaluated according to the CML-IA method, aiming to establish the environmental sustainability of the green synthesis compared to the coprecipitation method.^40^ CML-IA is a problem-oriented method that allows performing a quantitative, comparative, and comprehensive analysis of results.^51^ This method simplifies the interpretation of the environmental impacts in the cause–effect chain, reducing the uncertainty of the LCA study using midpoint impact categories.^52^ The geographic location scope was Cartagena (Bolívar), Colombia, which was used in this LCA study and to establish the contributions in the LCI.

#### 4.2.1 Goal and Scope Definition

**Scheme 1:**
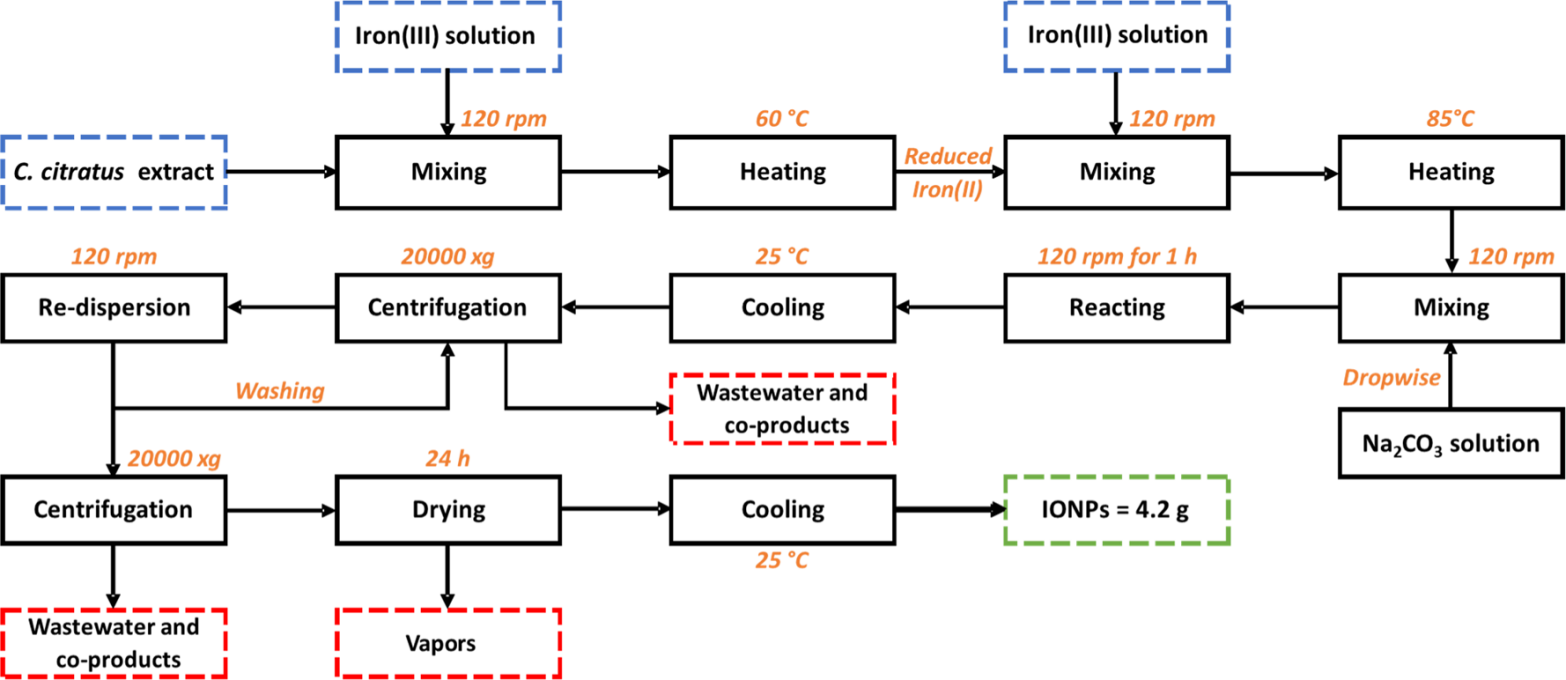
Block Diagram of the Green Synthesis of IONPs Process includes mixing, reaction, and purification stages

**Scheme 2:**
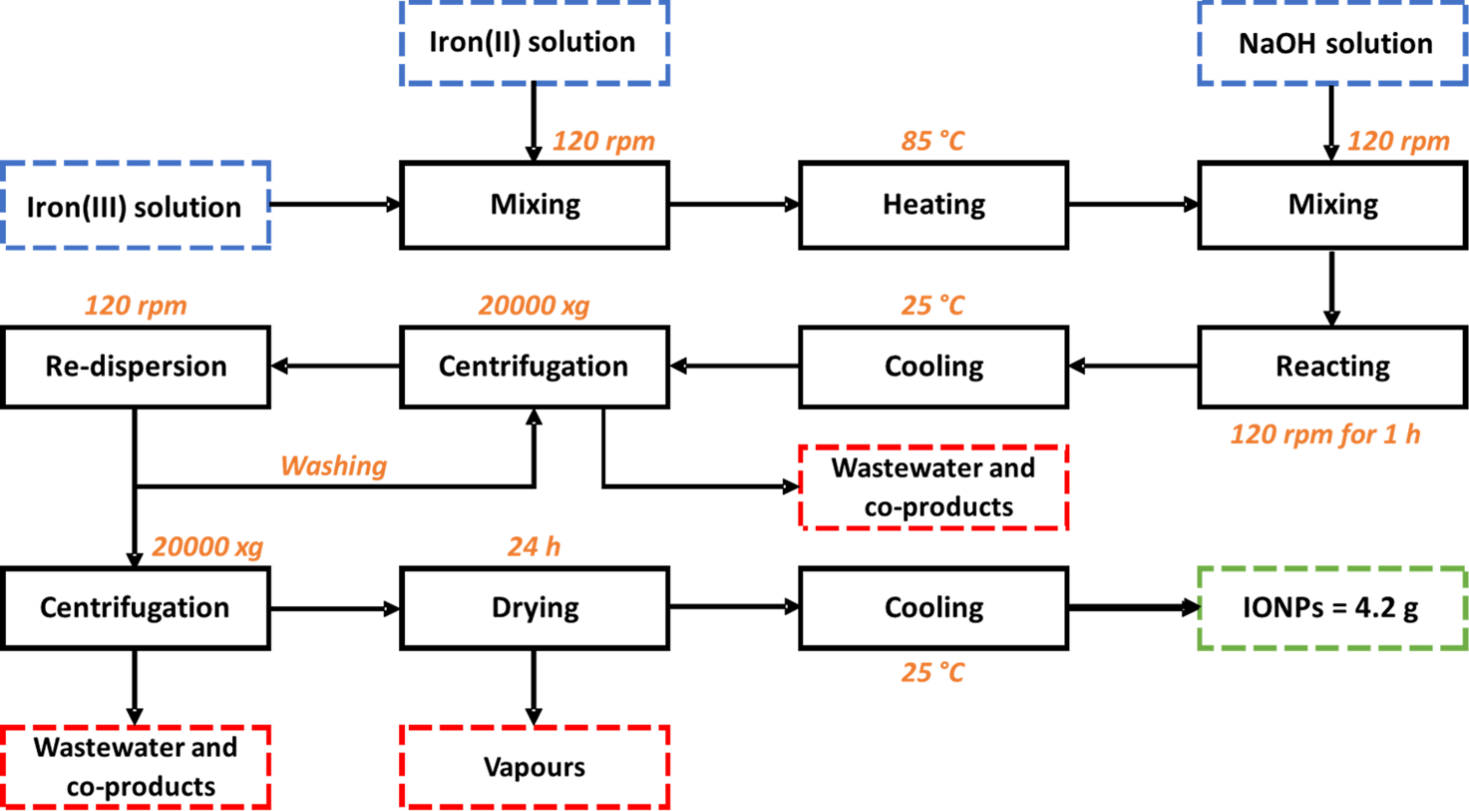
Block Diagram of the Coprecipitation Method This process excludes a mixing and a heating stage compared to the green synthesis

The LCA was performed based on a cradle-to-gate approach, including the quantification of the environmental impacts generated by the production of IONPs *via* the green synthesis and the coprecipitation method. Scheme 1 describes the procedure of the green synthesis at a laboratory scale and detailed information related to the stages and their operational conditions. In the case of the coprecipitation method, Scheme 2 shows a shorter procedure since the green synthesis required additional mixing and heating stages for the reduction of iron(III) to iron(II). The goal and scope aim to determine the applicability of the green synthesis for further investigations, including the scaling up of this procedure for the large-scale production of IONPs by following market policies and requirements.^50^ In this study, the functional unit was defined as the production of 1 g of IONPs, allowing normalization and calculation of the environmental impacts for further comparison of other results reported in the literature.

#### 4.2.2 System Boundaries

**Scheme 3:**
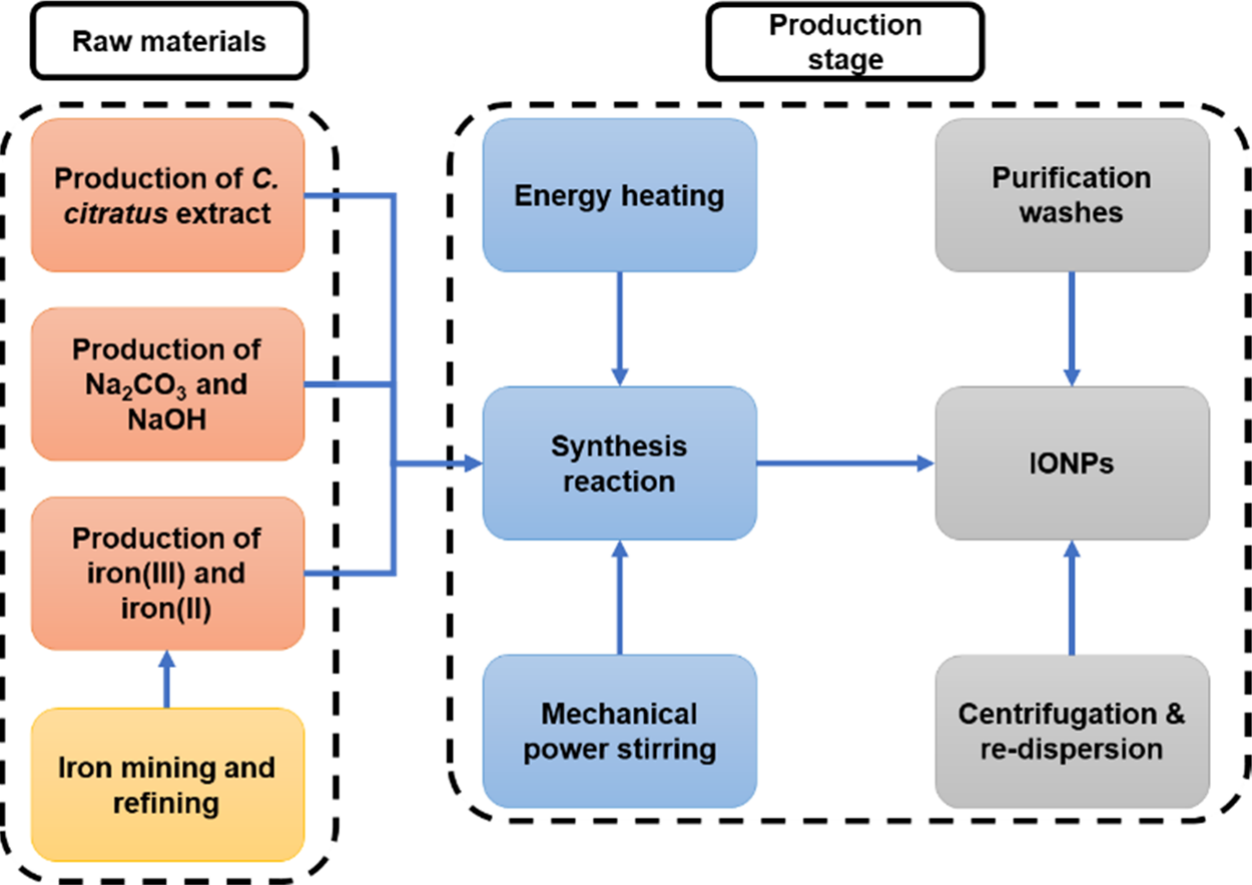
System Boundaries Following a Cradle-to-Gate Approach Three main phases are noted and applied for the green synthesis and coprecipitation method

The system boundaries delimit the phases included in the LCA by following a cradle-to-gate approach. Scheme 3 displays three main phases that include raw materials, production of IONPs, application, and final disposal. The first stage comprises the use of chemical and natural compounds, concerning anthropogenic activities, such as mining and refining, for their production. In the case of the production phase, reaction and purification stages are considered, in which input and output streams contain water, impurities, and coproducts. Additionally, energy consumption, wastewater, and emissions associated with these stages are included in the LCA of both methods. Finally, the phase related to the application and final disposal was discussed throughout the results, regarding raw materials and production of IONPs phases, due to the lack of information about nanomaterials in this field.

#### 4.2.3 Life Cycle Inventory

The LCI includes the primary data related to the input and output flows of the process, collected from the green synthesis and coprecipitation method at a laboratory scale. Additionally, and following the system boundaries described in Scheme 3, the LCI was also established according to the average data collected from the Ecoinvent 3.4 databases. Therefore, the LCI of the green synthesis and coprecipitation method are listed in Table 5. Here, the inventory consisted of input and output streams, in which the data was normalized to be analyzed and compared based on 1 g of IONPs. Four assumptions were considered in the green synthesis: (i) the *C. citratus* extract was mainly composed of water (98%) and citral (2%); (ii) all the iron(III) reacted and converted to IONPs; (iii) sodium chloride (NaCl) was formed as a coproduct during the reaction between iron(III) and Na_2_CO_3_, and (iv) carbon dioxide (CO) emission was produced during the conversion of Na_2_CO_3_ in the reaction. In the case of the coprecipitation method, the following assumptions were made: (v) all the iron(III) and iron(II) reacted and converted to IONPs, and (vi) NaCl was produced due to the use of NaOH in the coprecipitation of the iron chlorides.

**Table 5:** Process Inventory Including the Compounds Used in the Coprecipitation Method and the Green Synthesis^a^

coprecipitation method					green synthesis				
item	input (g)		output (g)		item	input (g)		output (g)	
	NN	N	NN	N		NN	N	NN	N
iron(III)	5.62	1.34	0.0	0.0	iron(III)	8.42	2.0	0.0	0.0
iron(II)	2.05	0.49	0.0	0.0	*C. citratus*	35.5	9.52	35.5	9.52
NaOH	4.0	0.95	4.0	0.95	Na_2_CO_3_	7.95	1.89	7.95	1.89
ethanol	189	45.1	189	45.1	ethanol	189	45.1	189	45.1
water	79	174	729	174	water	740	176	740	176
CO_2_	0.0	0.0	0.0	0.0	CO_2_	0.0	0.0	3.28	0.78
NaCl	0.0	0.0	3.32	0.79	NaCl	0.0	0.0	3.32	0.79
IONPs	0.0	0.0	4.2	1.0	IONPs	0.0	0.0	4.2	1.0
energy (kWh)	35.0	8.3	0.0	0.0	energy (kWh)	42.6	10.1	0.0	0.0

a (NN = not normalized, N = normalized)

The green synthesis and coprecipitation method required electricity generation for the thermal energy consumption in the reaction and purification (oven) stages. The equipment needed to perform the laboratory-scale methods is shown in Table 6, in which the total energy consumption was calculated, giving a value of 43.65 kWh. The quantification was performed using the power of each equipment and the operation time in the green synthesis and coprecipitation method. The separation of the IONPs in the coprecipitation method was assumed to be through magnetic separation, instead of using ultracentrifugation as in the green synthesis.

**Table 6:** Energy Consumption of Each Equipment^a^

	kW	co-precipitation		green synthesis	
equipment		time (h)	kWh	time (h)	kWh
heating plate	0.9	1.3	1.1	2.5	2.1
ultracentrifuge (20,000 g force)	3.8	0.0	0.0	1.9	7.3
rotator for stirring	0.1	2.3	0.3	4.3	0.6
electric oven	1.4	24.0	33.6	24.0	33.6
total	6.2	27.6	35.0	32.7	43.6

a Total Energy Required for the Green Synthesis was Higher due to the Use of Ultracentrifugation for the Separation of IONPs

#### 4.2.4 Environmental Impacts

The environmental impacts were established according to the CML-IA method, in which 11 categories were evaluated and are shown in Table 7. These impact categories were divided into three midpoints, including human health, ecosystem quality, and resource depletion. Human health represents the illnesses caused as a result of environmental deterioration, ecosystem quality involves the impacts on the fauna and flora, and resource depletion is related to the use of renewable and nonrenewable resources and their availability for the next generations.^53^ Here, the normalization of the environmental impacts allowed identifying and comparing the major contribution of both methods. The comparison was performed by dividing the characterization factors by the sum of the indicators per impact category, in which the results were given in neutral global units.^50^

**Table 7:** Environmental Impact Categories. Human Health, Ecosystem Quality, and Resource Depletion Midpoints Were Evaluated Using the CML-IA Method^54^

impact category	Abbreviation	dimension
abiotic depletion	AD	kg Sb eq
abiotic depletion (fossil fuels)	AD-ff	MJ
global warming potential	GWP	kg CO_2_ eq
ozone layer depletion	ODP	kg CFC-11 eq
human toxicity	HT	kg 1,4-DB eq
freshwater aquatic ecotoxicity	FAE	kg 1,4-DB eq
marine aquatic ecotoxicity	MAE	kg 1,4-DB eq
terrestrial ecotoxicity	TE	kg 1,4-DB eq
photochemical oxidation	PO	kg C_2_H_4_ eq
terrestrial acidification	TA	kg SO_2_ eq
Eutrophication	EP	kg PO_4_ eq

Considering the environmental impact results, a sensitivity analysis was performed for both the coprecipitation method and green synthesis based on the electricity in the LCI. Accordingly, the consumption of energy (electric and heat) was evaluated in three different scenarios: the base scenario consisted of the actual LCA study and a second and third scenario increased the consumption of energy by 50 and 100%, respectively. The results were normalized for a better comparison of the environmental impacts. Additionally, the uncertainty analysis was also implemented for verifying the probabilistic behavior of the process inventory data in the coprecipitation method and green synthesis. The uncertainty analysis was completed to verify the robustness of the results using Monte Carlo simulations.
